# Supplementary figures and images for: Autonomous epithelial folding induced by an intracellular mechano–polarity feedback loop
Source: PLoS Comput Biol. 2021 Dec 6;17(12):e1009614. doi: 10.1371/journal.pcbi.1009614 (PMC8675927; doi:10.1371/journal.pcbi.1009614)

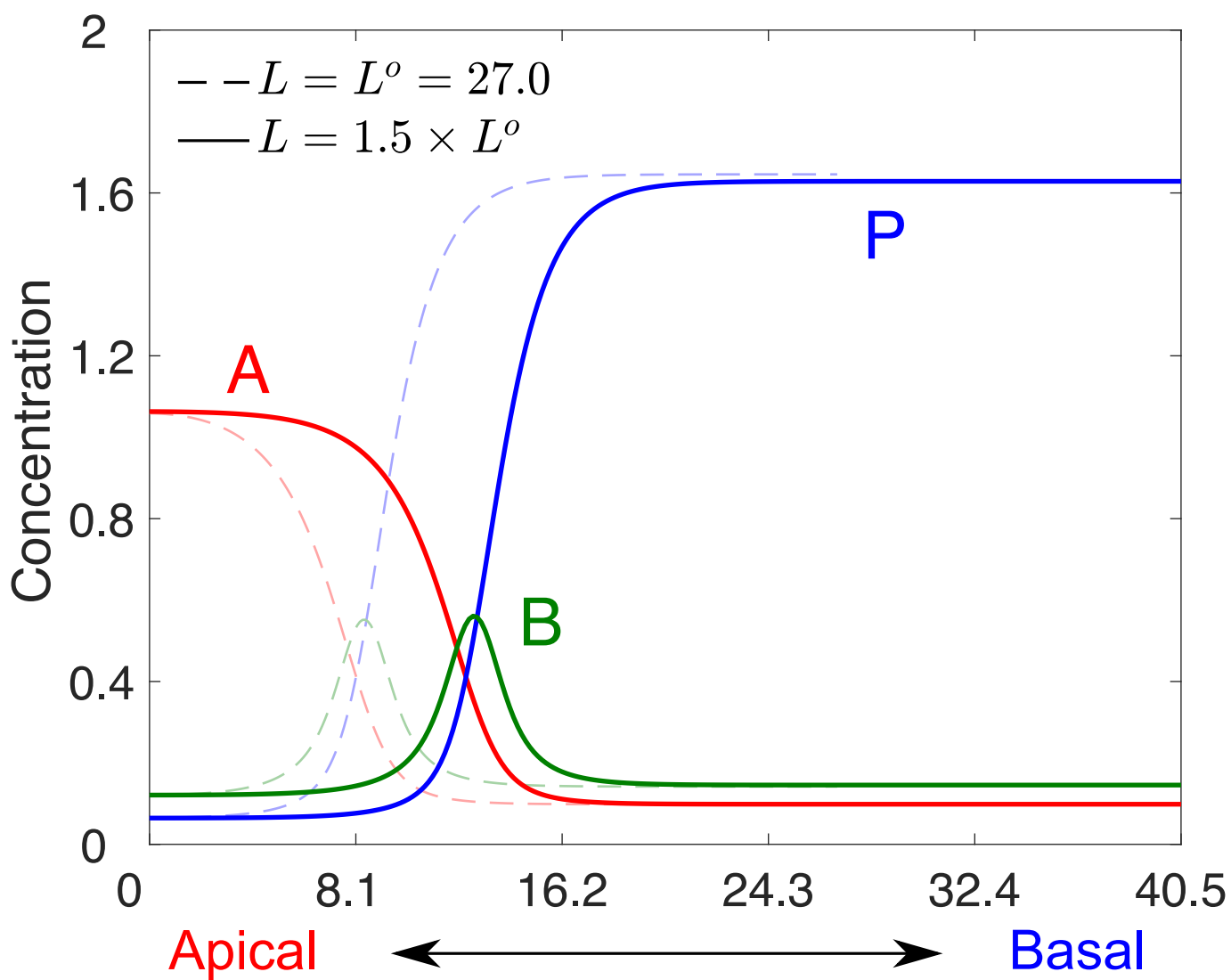

Supplement: S1 Fig — Note that the polarity distribution is plotted on the scale of absolute value of membrane location in contrast to Fig 3B where the membrane location is scaled by L. (PDF) [file pcbi.1009614.s001.pdf]

# A

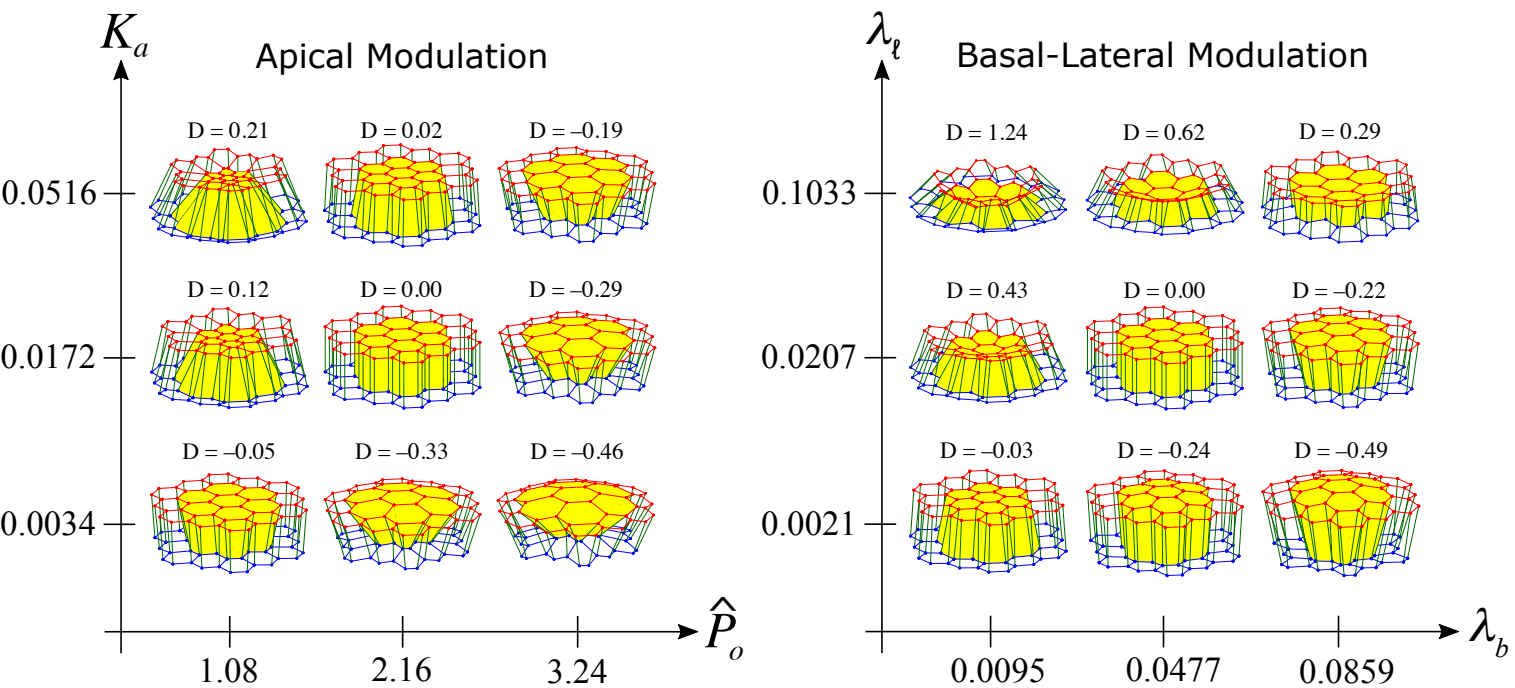

# B

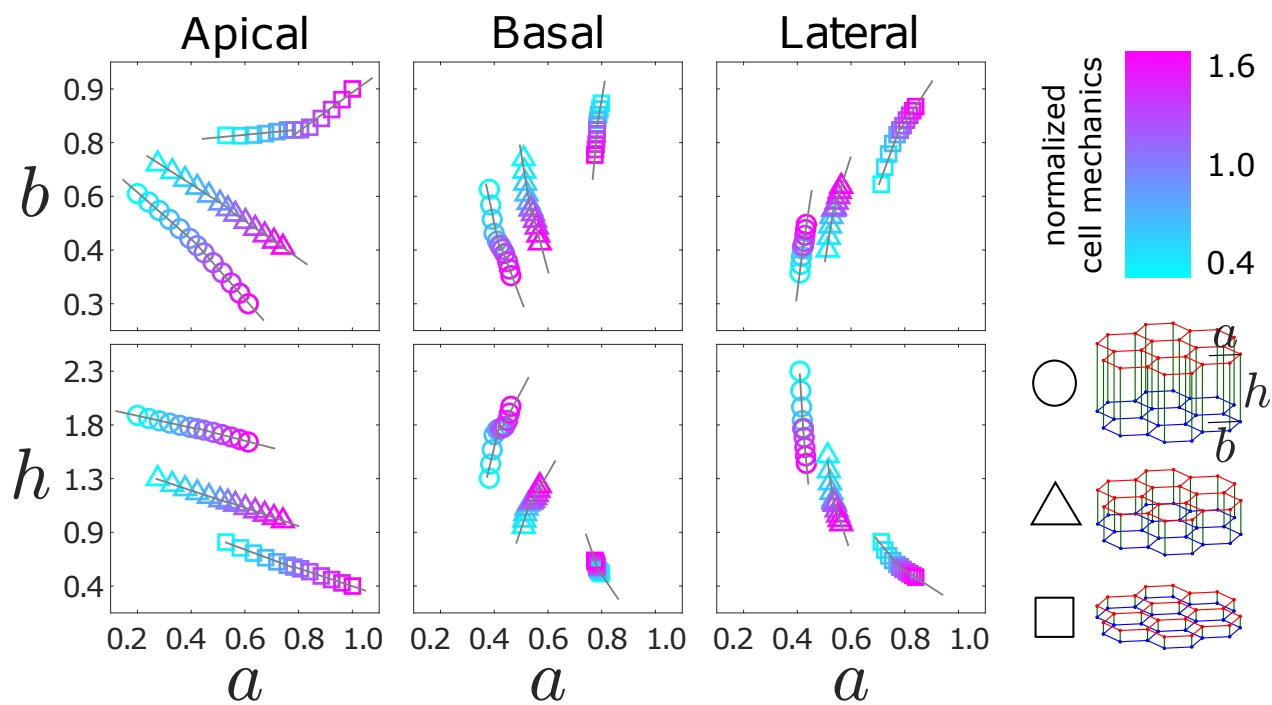

Supplement: S2 Fig — (A) A folded tissue structure forms when the apical mechanics, Ka and P^o (left), or the basal–lateral mechanics, λb and λℓ (right), of a local cell population (yellow) is modulated. The fold depth D is calculated as the difference between the highest and the lowest vertices on the apical surface, where D > 0 (D < 0) indicates inward (outward) folding. (B) Cell deformation patterns in response to modulation of the apical (left column), basal (middle column), or lateral cell mechanics (right column). For columnar (◯; a/h = 0.25), cuboidal (△; a/h = 0.50), and squamous (☐; a/h = 1.50) shapes, basal length b (top) and cell height h (bottom) are plotted against apical length a at the steady state for different values of mechanical parameters. The color bars indicate the relative deviation of parameter values from those of the flat epithelium, where the mechanics P^o, λb and λℓ are either increased or decreased for all the cells in the epithelium. The solid lines are linear regression lines that serve as a visual guide. (PDF) [file pcbi.1009614.s002.pdf]

control

packing topology

packing topology  
boundary restrictions

initial

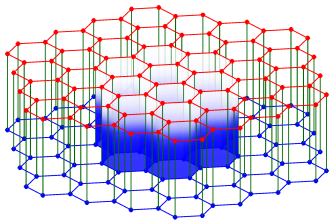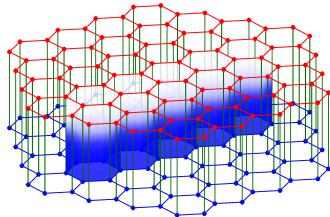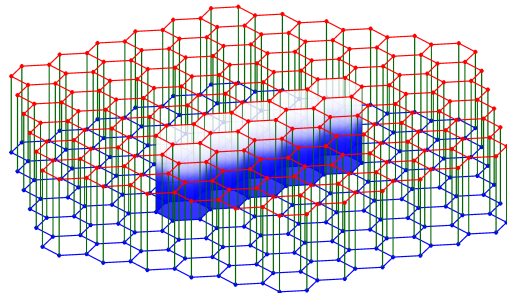

final

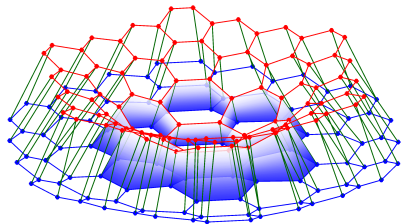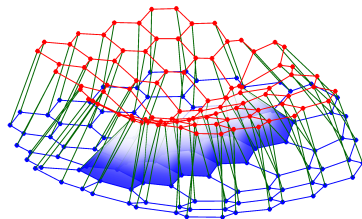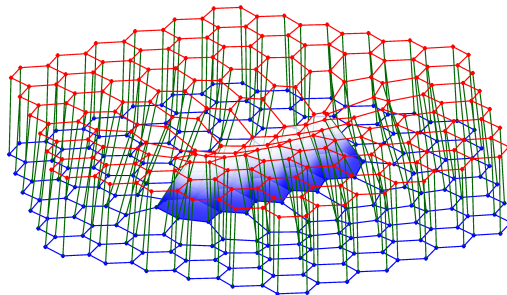

Supplement: S3 Fig — The initial (top row) and final homeostatic tissue shapes (bottom row) with the graded color showing P concentration (see color code in Fig 4B). Compared to the default concentric packing of the initiating cells (left column), a line of initiating cells results in the formation of a furrow-like fold (middle column). The fold formation is robust against boundary restrictions, where the movement of the outer boundary cells is confined to a horizontal plane, perpendicular to the apical–basal axis (right column). The parameter value cio={1.0,1.0,5.5,5.5} and others given in Table 1 were used in the simulations. (PDF) [file pcbi.1009614.s003.pdf]

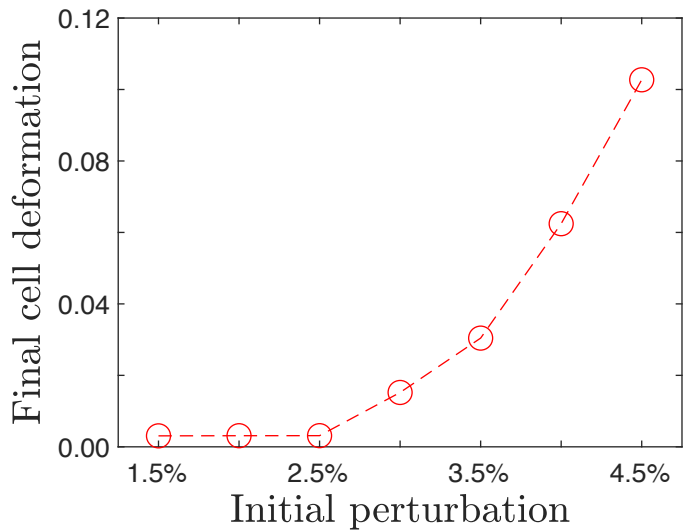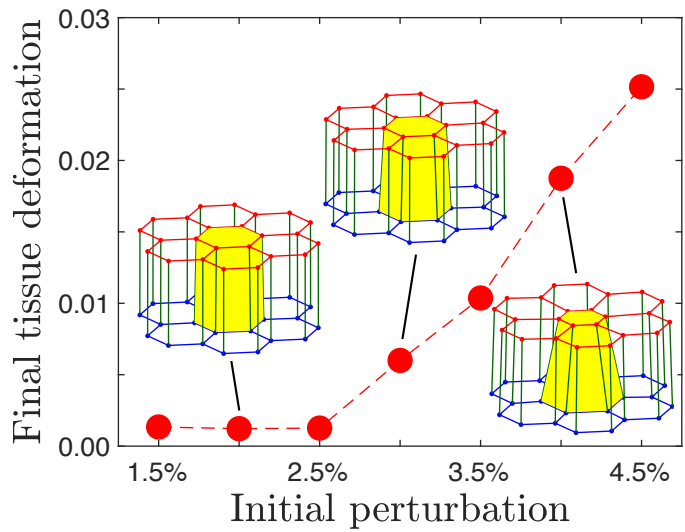

Supplement: S4 Fig — Insets in the right column show 3D visualization of the final steady-state tissue morphology following the initial biochemical perturbations. See Materials and methods for details of the analysis. (PDF) [file pcbi.1009614.s004.pdf]
